# Neurotrophic Factor Levels in Preterm Infants: A Systematic Review and Meta-Analysis

**DOI:** 10.3389/fneur.2021.643576

**Published:** 2021-04-01

**Authors:** Francieli Cristina Krey, Bruna Alvim Stocchero, Kerstin Camile Creutzberg, Bernardo Aguzzoli Heberle, Saulo Gantes Tractenberg, Li Xiang, Wei Wei, Bruno Kluwe-Schiavon, Thiago Wendt Viola

**Affiliations:** ^1^Developmental Cognitive Neuroscience Lab (DCNL), Graduate Program in Pediatrics and Child Health—School of Medicine, Pontifical University Catholic of Rio Grande Do Sul (PUCRS), Porto Alegre, Brazil; ^2^Department of Pharmacological and Biomolecular Sciences, University of Milan, Milan, Italy; ^3^DCNL, PUCRS, Graduate Program in Psychology—School of Health Sciences, Porto Alegre, Brazil; ^4^Neuroepigenetic Research Lab, Zhongnan Hospital of Wuhan University, Wuhan, China; ^5^School of Psychology, Psychology Research Centre, University of Minho, Braga, Portugal; ^6^Brain Institute of Rio Grande do Sul (BraIns), Pontifical Catholic University of Rio Grande do Sul, Porto Alegre, Brazil

**Keywords:** neurotrophic factors, brain derived neurotrophic factor, neurotrophins, preterm, infants, biomarkers

## Abstract

**Objectives:** Through a systematic review and meta-analysis of the literature we aimed to compare the levels of BDNF, NGF, NT-3, NT-4, and GDNF between human term and preterm infants, and investigate factors implicated in the variability of effect size estimates.

**Methods:** The analysis was performed in three online databases, MEDLINE Complete, PsycINFO, and CINAHL. A random effects model was used to calculate the standardized mean difference (SMD) of neurotrophic factor levels in preterm infants vs. term within a 95% confidence interval (CI). To explore sources of heterogeneity meta-regression models were implemented.

**Results:** Sixteen studies were included in this meta-analysis. A combined sample of 1,379 preterm and 1,286 term newborns were evaluated. We identified significant lower BDNF (SMD = −0.32; 95% CI: −0.59, −0.06; *p* = 0.014) and NT-3 (SMD = −0.31; 95% CI: −0.52, −0.09; *p* = 0.004) levels in preterm compared to term infants. No significant difference was observed in NGF and NT-4 levels between groups. Given that only two effect sizes were generated for GDNF levels, no meta-analytical model was performed. Meta-regression models revealed sample type (placental tissue, cerebrospinal fluid, peripheral blood, and umbilical cord blood) as a significant moderator of heterogeneity for BDNF meta-analysis. No significant associations were found for gestational week, birth weight, and clinical comorbidity of newborns with effect sizes.

**Conclusions:** Our findings indicated that lower BDNF and NT-3 levels may be associated with preterm birth. Future studies with larger samples sizes should investigate neurodevelopmental manifestations resulting from neurotrophic factor dysregulation among preterm infants.

## Introduction

Premature birth is defined as infant delivery before 37 weeks of gestation. Worldwide, it has been estimated that 15 million premature births occur annually (1). This is one of the most common risk factors for infant mortality and it has been associated with long-term adverse consequences for the development of central and peripheral nervous systems (2). There is a growing body of research on biomarkers that might help identify an increased risk for negative neurodevelopmental outcomes among premature children (3).

Neurotrophic factors (NFs) are molecules that have different and essential functions in central and peripheral nervous tissues, mediating cell development, survival, and maintenance. NFs play critical roles in both of the development of neurons as well as the plasticity and functionality of mature cells, including axonal growth and orientation, dendritic development, and synaptic plasticity (4). Substantial evidence has shown that NFs could directly interact with neurotransmitter systems that are involved with motor and cognitive functioning (5, 6). NFs can be grouped into two main families: neurotrophins and glial cell-derived neurotrophic factor (GDNF) (7). Neurotrophins include primarily the nerve growth factor (NGF), brain-derived neurotrophic factor (BDNF), neurotrophin-3 (NT-3), and neurotrophin-4 (NT-4) (8). NFs are not only produced by nerve cells, but are also found in immune cells, adipocytes, endocrine, and endothelial cells, playing an important role for the integrative functioning of these biological systems (9). From early brain ontogenesis to the adulthood, altered expression of NFs has been associated with neurological and psychiatric illnesses (10). In light of that, there is a growing and promising research field assessing the levels of NFs in peripheral samples (e.g., blood, cerebrospinal fluid, immune cells, etc.) as diagnostic and prognostic markers of different brain-related conditions in children with premature birth (11). Although systematic reviews about the topic have been published (12), to the best of our knowledge no meta-analysis has attempted to synthesize the research literature so far.

Therefore, through a systematic review and meta-analysis of the literature, the present study aimed to compare the levels of BDNF, NGF, NT-3, NT-4, and GDNF between term and preterm infants, and investigate factors implicated in the variability of effect size estimates, such as sample type, gestational weeks, birth weight, and clinical comorbidity of newborns with preterm birth. The main hypothesis is that premature birth would be associated with altered levels of at least one NF, which may be an indicative of a potential biomarker for the negative neurodevelopmental consequences of premature birth, allowing new perspectives for early and preventive interventions.

## Methods

### Search Strategy

The search was performed in three online databases, MEDLINE Complete, PsycINFO, and CINAHL, in January 2020 and then updated in November 2020. There was no restriction regarding language or publication date of the studies. A filter to select only studies with humans was used. The PECO framework was implemented (1) Patient: Newborns; (2) Exposure: Preterm; (3) Comparison: Term; (4) Outcome: Neurotrophic Factors; and guided the search strategy using the following terms: [(neurotrophic factors) OR (NGF) OR (BDNF) OR (NT3) OR (NT4) OR (NT4/5) OR (GDNF) OR (CDNF) OR (MANF)] AND [(preterm) OR (premature birth)] Filters: Humans. In the research strategy, we also included the cerebral dopamine neurotrophic factor (CDNF) and mesencephalic astrocyte-derived neurotrophic factor (MANF), which consist in a novel family of NFs (https://pubmed.ncbi.nlm.nih.gov/20186704/).

### Screening and Eligibility

The selection was done in three phases. Initially, all searched results from all databases were merged and the duplicates excluded (based on titles and abstracts). Subsequently, references were imported to Rayyan (https:// rayyan.qcri.org), a free web application for management of systematic reviews. Then, in the second step, titles and abstracts were screened and any study design using a quantitative framework for data analysis (e.g., randomized controlled trials, cross-sectional studies, longitudinal studies, retrospective studies, case-control studies, etc.) was initially included. In addition, any study that clearly did not assess NFs in preterm infants were excluded in this step. Studies where we could not tell whether NFs were assessed through title/abstract analysis, were included for the next phase of eligibility. In the third phase, the full-text articles were assessed for eligibility based on seven exclusion criteria: (1) Did not assess NFs; (2) had no specific data on term infants; (3) had no specific data on preterm infants; (4) had no available data on between group comparisons regarding NFs; (5) had only maternal blood analysis without newborn specific data; (6) had evaluated NFs but had no specific data for BDNF, NGF, NT-3, NT-4, GDNF, CDNF, AND MANF; (7) manuscript withdrawn. Both selection phases were performed independently by two authors (FK and BAS) using the Rayyan Software (13). Any disagreements about study inclusion or exclusion during this process were resolved in consensus discussions with two other authors (SGT and TWV).

### Data Extraction

The following data were extracted from all included studies by two independent authors (FCK, BS, and BKS): “first author,” “publication year,” “study design,” “sample size,” “clinical conditions of preterm newborns,” “gestational weeks,” “birth weight,” “NF method of assessment,” “analyzed sample type,” “timeframe of assessment,” “qualitative main findings.” The mean and the standard deviation (SD) were recorded for NFs from the term and preterm groups. If instead of mean and SD, other values were reported (median, standard error, or interquartile range), the mean and SD were estimated as follows: (1) median as mean; (2) standard error multiplied by the square root of sample size, as SD; and (3) interquartile range divide by 1.35, as SD. Moreover, if the article presented the data only in graphs and not as text, the data was extracted using the software Getdata (version 2.26.0.20, © S.Fedorov).

### Methodological Quality Assessment

To assess the methodological quality and risk of bias in the included studies, an adapted version of the Newcastle-Ottawa Scale (NOS) was used (14). The NOS consists of nine items that detect bias related to selection (representativeness of the sample, sample size, non-respondents, ascertainment of exposure), comparability (controlling for one or more confounding factors) and outcome (information on gestational age, weight, and APGAR score). A study with score 128 of “yes” has a low risk of bias while a score of “no” indicates a high risk of bias.

### Data Analysis

The statistical analyses were performed in two steps. Initially, to compare the levels of BDNF, NGF, NT-3, NT-4, and GDNF between term and preterm infants, independent random effects meta-analytical models were performed based on the standardized mean differences (SMD) calculated by use of Cohen's *d*, within a 95% confidence interval (CI). Given that only two effect sizes were generated for GDNF levels, no meta-analytical model was performed for this NF. *Q* statistic was used to test the existence of heterogeneity and *I*^2^ to assess the proportion of total variability due to heterogeneity.

To achieve the second aim of the study and explore sources of heterogeneity in statistically significant meta-analyses univariate meta-regression models were implemented. We included the following potential sample-related moderators: gestational weeks, birth weight, and the presence of clinical comorbidities among preterm (e.g., preeclampsia). Additionally, the following methodological moderator was considered: analyzed sample type (e.g., newborn blood, umbilical cord, placental tissue, or liquor).

Furthermore, in both meta-analytic and meta-regression procedures, if studies presented data of two distinct samples (e.g., preterm and preterm with a clinical condition), both sets of data were included, but with the same identification and thus, the same random effects. Because these sets of data “shared” the same term control group, we split into two groups with smaller sample sizes (i.e., half). By dividing the sample size of the shared control group by the number of comparisons with independent clinical groups from the same study, we decreased the likelihood of overweighting the effect sizes (15).

Finally, outliers and influential cases were detected using the externally standardized residuals, DFFITS values, Cook's distances, covariance ratios, leave-one-out estimates of the amount of heterogeneity, leave-one-out heterogeneity test statistics, hat values, and weights. Egger's regression test for funnel plot asymmetry was performed for publication bias analysis. All statistical analyses were performed using the package “metaphor” (version 2.4-0) from the open-source statistical software R (version 4.0.0) (16).

## Results

The initial database search yielded 249 studies. After excluding duplicate records (*n* = 23), we screened 226 studies through the review of title and abstract. After that 174 studies were excluded and the remainder (*n* = 52) were full text reviewed applying the exclusion criteria. Following the application of these criteria, a total of 14 studies were included. After the search update two additional studies were included in this review. The 16 reviewed studies combined for a sample of 1,379 preterm and 1,286 term newborns. Figure S1 displays the flowchart of this systematic review.

### Characteristics of Studies

The range of publication year of the included studies was 2004–2020 (17–32). Fifty-six percent (*n* = 9) were case-control studies, 19% (*n* = 3) were cohort studies, 19% (*n* = 3) were cross-sectional studies, while one study was a randomized clinical trial (Table 1). In addition to comparisons between term and preterm newborns, 56% (*n* = 9) of the studies included a third group in the analyses (e.g., very preterm, preterm with preeclampsia, preterm with bronchopulmonary dysplasia, preterm receiving treatment with steroids, or magnesium sulfate). According to the NOS (Table 2), most studies assessed had a score >6 (69%, *n* = 11), and were considered as “low risk of bias.” Only five studies were considered as “high risk of bias.”

**Table 1:** Characteristics of the included studies.

**Reference**	**Study design**	**Participants**	**GA (weeks)**	**Weight (kg)**	**Neurotrophic factors**	**Evaluation methods**	**Main findings**
(30)	Case-control	Total = 79 **1**. Term = 52 **2**. Preterm = 27	**1**. 38.4 **2**. 33.0	**1**. 3.17 **2**. 2.11	BDNF, GDNF, NGF, and NT-4 protein	Collection of umbilical cord blood at birth. The analysis was made with Luminex xMAP technology.	• Levels of BDNF were significantly lower in preterm neonates when compared to term infants • Levels of GDNF were significantly higher in preterm neonates compared to term infants • There was no statistical difference in levels of NGF and NT-4 between groups.
(32)	Prospective Cohort	Total = 85 **1**. Term = 14 **2**. Preterm with Seizure = 13 **3**. Preterm without Seizure = 13	**1**. 38.3 **2**. 30.6 **3**. 30.9	**1**. 3.20**2**. 1.14**3**. 1.23	BDNF protein	ELISA were used to determine blood serum and CSF levels of BDNF. They were measured in the first 48 h of life.	• Levels of BDNF were significantly lower in preterm neonates when compared to term infants only in serum samples, but not in CSF samples • A tendency, with no statistical significance, for higher values for serum and CSF levels of BDNF in neonates with favorable than with unfavorable outcome regarding prognostic outcome, MRI/CT findings, a EEG back-ground activity and the need for assisted ventilation in NICU in overall study population was observed
(29)	Prospective cohort	Total = 171 **1**. Term = 47 **2**. Preterm without BPD = 92 **3**. Preterm with BPD = 32 BPD: broncho pulmonary dysplasia	**1**. 39.7 **2**. 33.9 **3**. 26.0	**1**. 3.59 **2**. 1.93 **3**. 0.80	BDNF and NGF protein	Collection of peripheral blood samples within the first 48 h after birth. Serum samples were analyzed using immunoassays	• BDNF and NGF levels were significantly lower in preterm infants with BPD as compared to preterm infants without BPD and term infants • Lower levels of serum BDNF were significantly associated with higher risk of BPD • NGF levels measured at birth had highly significant positive correlation with language and cognitive outcomes at 24 months of life, but no significant correlation with motor development • BDNF levels had no significant correlation with neurodevelopmental outcomes
(31)	Cross-sectional	Total = 71 **1**. Term = 30 **2**. Term with preeclampsia = 18 **3**. Preterm with preeclampsia = 23	**1**. 39.3 **2**. 39.3 **3**. 33.8	**1**. 3.0 **2**. 2.9 **3**. 1.7	BDNF and NGF mRNA	Placental tissue samples were collected immediately after delivery, from four different regions: Central maternal (CM), Central fetal (CF), Peripheral maternal (PM) and Peripheral fetal (PF). RT-qPCR was used for analyses	• In the CM region, BDNF levels were significantly lower in preterm infants with preeclampsia to term infants without preeclampsia • No group differences were found for NGF levels
(27)	Case-control	Total = 37 **1**. Term = 20 **2**. Preterm = 17	**1**. 39.8 **2**. 27.1	Missing	BDNF, NGF and NT-3 protein	Collection of CSF samples. Custom antibody array for 50 human analytes was used	• BDNF levels were significantly higher in preterm infants as compared to term infants • No group differences were found for NGF levels
(28)	Case-control	Total = 20 (umbilical cord analysis) **1**. Term = 14 **2**. Preterm = 6 Total = 58 (CSF analysis) **(A)** Term = 35 **(B)** Preterm = 23	**1**. Varies from 38 to 41 **2**. Varies from 30 to 35 **(A)** 38.0 **(B)** 27.7	**1**. 3.28**2**. 2.01**(A)** 3.09**(B)** 1.20	GDNF protein	Collection of CSF and umbilical cord blood samples. ELISA kit for analyses	• Cord blood GDNF levels were significantly higher in preterm newborns when compared to term newborns • CSF-GDNF levels were similar between groups • CSF-GDNF levels trended, with no statistical significance, higher in premature infants with post hemorrhagic ventriculomegaly as compared to those who had no significant abnormality on head ultrasound scans
(26)	Case-control	Total = 248 **1**. Term = 129 **2**. Late preterm unexposed to steroids = 51 **3**. Late preterm exposed to steroids = 68	**1**. 39.0 **2**. 35.0 **3**. 34.0	**1**. 3.33**2**. 2.4**3**. 2.27	BDNF, NT3, NT4 and NGF protein	Collection of umbilical cord blood serum samples. The analysis was made using ELISA kits	• NT-4 and NGF concentrations were significantly lower in late preterm infants compared to term newborns • NT3 concentrations were significantly lower in late preterm infants exposed to steroids as compared to unexposed late preterm infants and term infants
(25)	Cross-sectional	Total = 97 **1**. Term = 50 **2**. Term with preeclampsia = 21 **3**. Preterm with preeclampsia = 26	**1**. 38.9 **2**. 38.6 **3**. 34.2	**1**. 2.9**2**. 2.7**3**. 1.7	BDNF and NGF protein	Placental tissue samples were collected immediately after delivery, from four different regions: Central maternal (CM), Central fetal (CF), Peripheral maternal (PM) and Peripheral fetal (PF). Emax Immunoassays were used for analyses	• NGF levels were significantly higher in the PF, CM, and PM regions in preterm preeclampsia group as compared to term infants • No differences were found for BDNF levels • Negative significant association of NGF levels with baby weight were found • Positive significant association of NGF and baby systolic blood pressure were found
(23)	Randomized control trial	Total = 72 **1**. Term = 24 **2**. Preterm with MgSO_4_ = 24 **3**. Preterm without MgSO_4_ = 24	• 39.5 • 33.4 • 33.2	• Missing • 2.22 • 2.15	BDNF protein	Collection of umbilical cord blood samples shortly after birth. The analysis was made with ELISA	• BDNF levels in preterm with antenatal MgSO_4_ were significantly higher when they were compared to premature preterm without antenatal MgSO4 • BDNF levels in preterm without antenatal MgSO_4_ were significantly lower than in full-term infants • There was no significant difference in BDNF levels between preterm with MgSO4 and full-term infants
(24)	Cross-sectional	Total = 201 **1**. Term = 95 **2**. Term with preeclampsia = 60 **3**. Preterm with preeclampsia = 46	**1**. 39.1 **2**. 38.7 **3**. 34.2	**1**. 2.9**2**. 2.7**3**. 1.9	BDNF protein	Collection umbilical cord blood samples at the time of delivery. The analysis was made with Emax Immunoassay	• BDNF levels were significantly higher in newborns with preeclampsia (term and preterm) when compared to normotensive group • Among the preeclampsia groups, BDNF levels in the preterm group was significantly lower as compared to the term group
(21)	Case-control	Total = 191 **1**. Term = 95 **2**. Preterm = 96	**1**. 39.2 **2**. 34.3	**1**. 2.92 **2**. 2.02	BDNF protein	Collection of umbilical cord blood samples during delivery. The analysis was made with BDNF Emax Immunoassay	• BDNF levels were significantly lower in preterm deliveries as compared to term deliveries • A positive significant correlation between BDNF levels and gestational weeks was observed • No significant correlation was found between BDNF and placental weight
(22)	Case-control	Total = 190 **1**. Term = 94 **2**. Preterm = 96	**1**. 39.1 **2**. 34.6	**1**. 2.94**2**. 2.15	NGF protein	Collection of umbilical cord blood samples during delivery. The analysis was made with NGF Emax immunoassay	• NGF levels were significantly lower in preterm group as compared to the term group • No significant correlation was found between NFG and birth outcomes
(20)	Case-control	Total = 927 **1**. Term = 439 **2**. Moderately Preterm = 372 **3**. Very Preterm = 116	**1**. 39.5 **2**. 35.5 **3**. 30.1	**1**. 3.25**2**. 2.55**3**. 1.53	BDNF, NT-3, and NT-4 protein	Collection of umbilical cord blood at delivery. The analysis was made with Luminex xMAP technology	• Concentrations of BDNF and NT-3 were significantly decreased in both preterm births (very preterm and moderately preterm) • Concentrations of NT-4 were not significantly different between gestational age groups
(19)	Case-control	Total = 160 **1**. Term = 82 **2**. Preterm = 52 **3**. Very preterm = 26	Missing	Missing	BDNF and NT-4 protein	Collection of residual newborn screening dried blood spots samples. All samples were drawn before 10 days of age. The analysis was made with Luminex xMAP technology	• BDNF levels were significantly decreased in preterm as compared with term neonates • Concentrations of NT-4 were not significantly different between gestational age groups
(18)	Case-control	Total = 183 **1**. Term = 50 **2**. Very preterm = 28	Missing	Missing	BDNF, NT-3, and NT-4 protein	Collection of blood spot samples. The analysis was made with ELISA kits	• BDNF levels were significantly lower in very preterm newborns as compared with term babies • No significant differences between groups was found for NT-3 and NT-4
(17)	Prospective cohort	Total = 45 **1**. Term = 30 **2**. Preterm = 15	**1**. 39.2 **2**. 29.4	Missing	BDNF protein	Collection of umbilical cord blood at delivery and of neonatal blood in the first and 4th day postpartum. BDNF was determined by ELISA	• Levels of BDNF were significantly higher in full-term babies when compared to pre-term babies • Levels of BDNF in preterm neonates in the 1st day postpartum had a significant increased as compared to concentration levels in the umbilical cord of preterm babies

*CSF, cerebrospinal fluid; kg, kilograms; ELISA, Enzyme-linked Immunosorbent assays*.

**Table 2:** Modified Newcastle–Ottawa Scale (NOS) for assessing the quality of studies in meta-analyses.

**Studies**	**Selection**				**Comparability**	Outcome^*^			**Total Quality Score**
**Reference**	**Is the Case Definition Adequate?**	**Representativeness of the Cases**	**Selection of Controls**	**Definition of Controls**	**Comparability of cases and controls**	**Gestational age**	**Weight**	**Apgar Score**	
(30)	1	0	1	1	0	1	1	1	6
(32)	1	0	1	1	1	1	1	1	7
(27)	1	0	1	1	0	1	0	0	4
(28)	1	0	1	1	2	1	1	0	7
(26)	1	0	1	1	2	1	1	1	8
(25)	1	0	1	1	2	1	1	0	7
(23)	1	0	1	1	2	1	1	0	7
(24)	1	0	1	1	2	1	1	0	7
(21)	1	0	1	1	2	1	1	0	7
(22)	1	0	1	1	2	1	1	0	7
(20)	1	1	1	1	2	1	1	0	8
(19)	1	1	1	1	2	0	0	1	7
(18)	1	0	0	1	0	0	0	0	2
(17)	1	0	0	1	0	1	0	1	4

* *Modified outcome items adapted for studies investigating samples of preterm infants*.

For the analysis of NFs, 25% (*n* = 4) studies collected peripheral blood samples from newborns, while 12% (*n* = 2) used placental tissue, 62% (*n* = 10) used umbilical cord blood and 12% (*n* = 2) analyzed cerebrospinal fluid samples. Most studies collected samples immediately after birth up to 48 h post-delivery (62%; *n* = 10). Protein levels were assessed by the majority of the studies, with one exception where gene expression analysis was performed. BDNF was evaluated in 81% (*n* = 13) of the studies, while NGF in 37% (*n* = 6), NT-3 in 19% (*n* = 3), NT-4 in 31% (*n* = 5), and GDNF in just two of the studies. Therefore, we did not perform a meta-analysis for GDNF data due to the small number of studies. Moreover, no studies investigated CDNF/MANF, therefore, these NFs were not included on both the systematic review and the meta-analysis. Detection methods were limited mostly to enzyme-linked immunosorbent assay (ELISA), Luminex xMAP technology, and Emax Immunoassays. More detailed information regarding the main findings of each study, NOS score, NF method of assessment, and sample specification are reported in Table 1.

### Preterm Effects on Neurotrophic Factors

**Figure 1:**
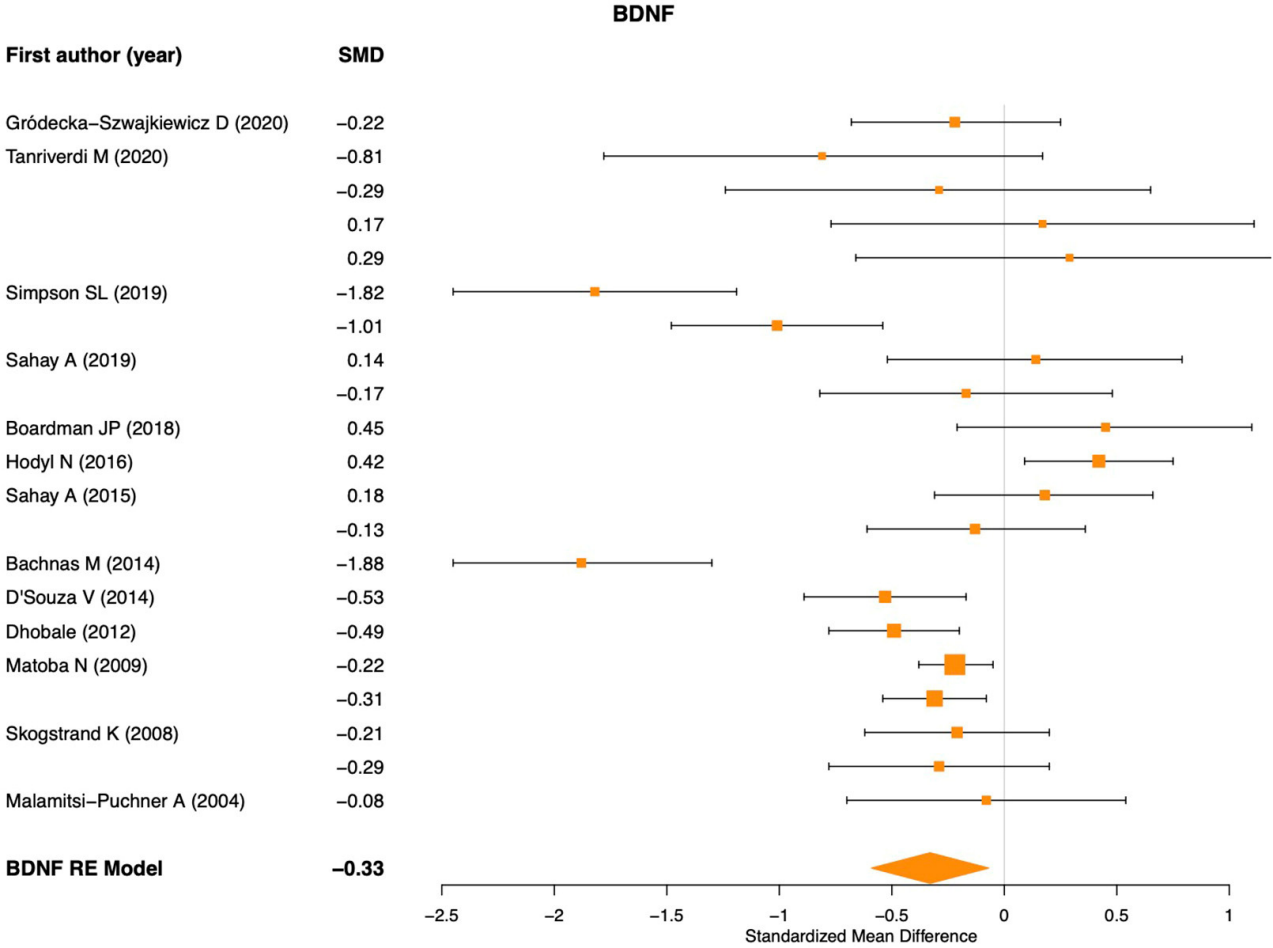
BDNF meta-analysis. RE, random effects model.

Influential case analysis detected one outlier (18), resulting in a total of 21 effect sizes used for BDNF meta-analysis. A significant effect was found showing lower BDNF levels in preterm newborns compared to term (SMD = −0.32; 95% CI: −0.59, −0.06; *p* = 0.014; Figure 1). This analysis revealed significant heterogeneity across studies (*Q*_*p*__−val_ < 0.001). Univariate meta-regression models revealed only sample type as a significant moderator of heterogeneity, showing higher BDNF levels in placental tissue (*p* < 0.041) and cerebrospinal fluid (*p* < 0.016) than in peripheral blood and umbilical cord blood samples. No significant associations were found for the gestational week, birth weight, and clinical comorbidity of newborns with BDNF effect sizes.

**Figure 2:**
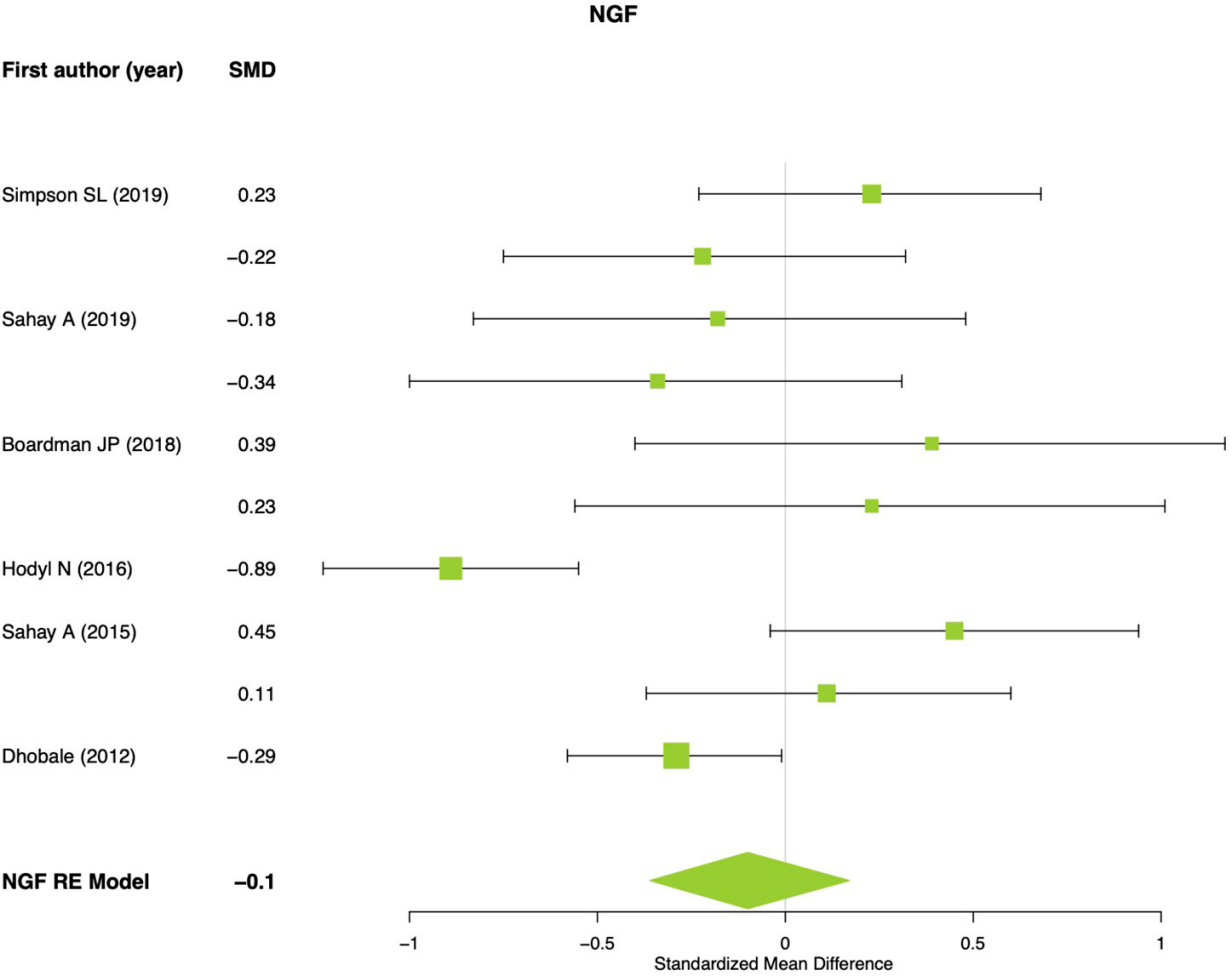
NGF meta-analysis. RE, random effects model.

**Figure 3:**
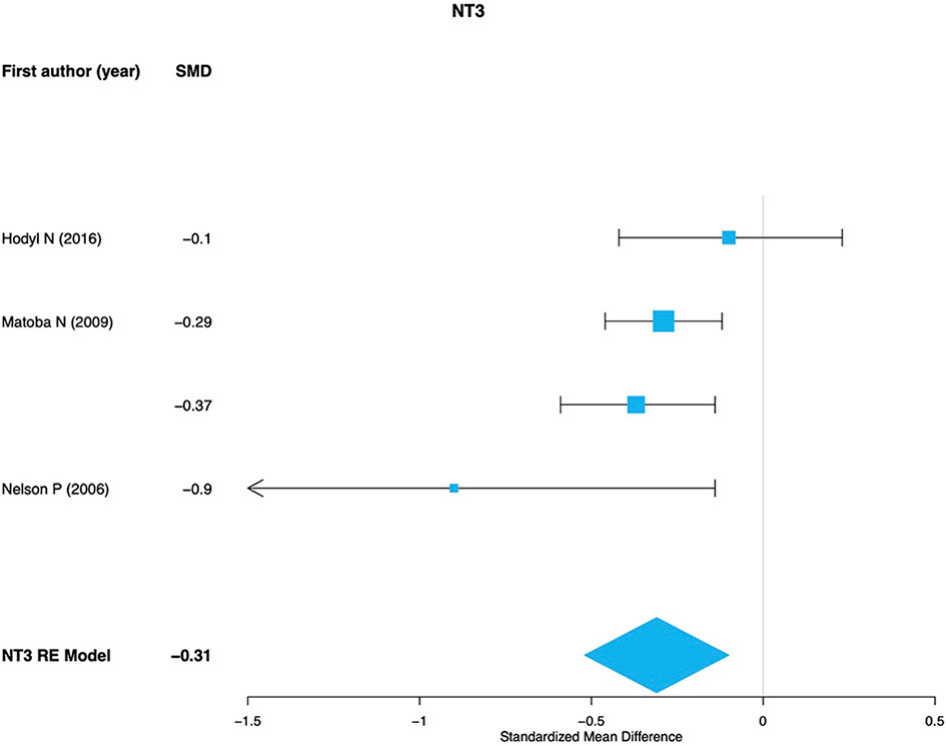
NT-3 meta-analysis. RE, random effects model.

**Figure 4:**
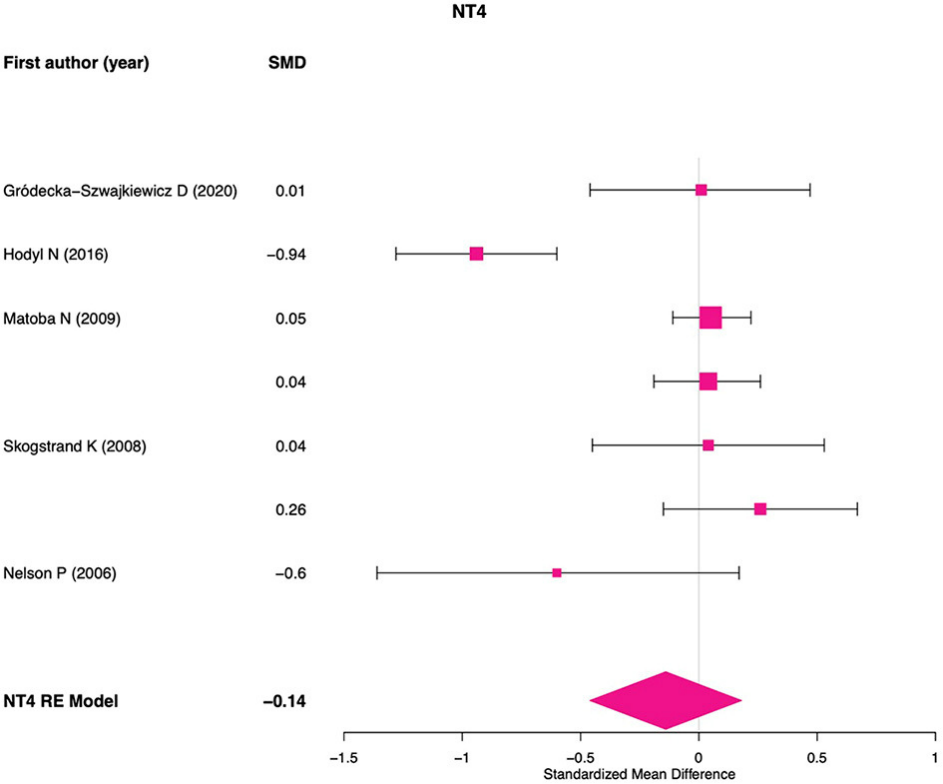
NT-4 meta-analysis. RE, random effects model.

For NGF meta-analysis 10 effect sizes were used. No significant differences were found between preterm and term newborns (SMD = −0.09; 95% CI: −0.36, 0.16; *p* = 0.477; *Q*_*p*__−val_ < 0.001; Figure 2). No significant or marginal associations were found for potential moderators. For NT-3 meta-analysis four effect sizes were used and a significant effect was found showing lower NT-3 levels in preterm newborns compared to term (SMD = −0.31; 95% CI: −0.52, −0.09; *p* = 0.004; *Q*_*p*__−val_ = 0.232; Figure 3). For NT-4 meta-analysis seven effect sizes were used and no significant differences were found between preterm and term newborns (SMD = −0.13; 95% CI: −0.45, 0.17; *p* = 0.389; *Q*_*p*__−val_ < 0.001; Figure 4). No significant or marginal associations were found for potential moderators regarding NT-4. Egger's regression test for funnel plot asymmetry did not show evidence of publication bias for any of the meta-analyses performed.

In addition to preterm and term comparisons performed in the meta-analyses, findings showing an association between NFs and clinical outcomes were reported in some studies (Table 1). Simpson et al. (29) found that lower levels of serum BDNF were significantly associated with a higher risk of bronchopulmonary dysplasia among preterm newborns. Furthermore, Dhobale et al. (21) showed a positive correlation between BDNF levels and gestational weeks. Regarding NGF, Simpson et al. (29) documented that the levels measured at birth had a highly significant positive correlation with language and cognitive outcomes at 24 months of life. Moreover, a positive association of NGF and baby systolic blood pressure was found by Sahay et al. (25). No statistically significant clinical correlation was observed for the remaining NFs among the reviewed studies.

## Discussion

We conducted a systematic review and meta-analytic study addressing the consequences of preterm birth on peripheral NF levels. Our findings revealed data supporting altered levels of specific NFs among preterm newborns, as indicated by decreased levels of BDNF and NT-3 when compared with term newborns. Our results also suggested that sample type (e.g., peripheral blood, cerebrospinal fluid, etc.) is an essential moderator of heterogeneity between studies assessing BDNF levels. No evidence for altered levels of NGF and NT-4 was found, while GDNF was not assessed due to the small number of studies measuring it. A qualitative synthesis of the studies, primarily through correlational analyses reported in the studies, suggests that BDNF and NGF are the main biomarkers associated with neurodevelopmental and clinical outcomes during the first stages of early development.

BDNF was the most often evaluated NF among the reviewed studies, in conformity with the neurodevelopmental disorder literature. Several studies have found that lower BDNF levels are associated with diseases diagnosed in youth (e.g., autism spectrum disorder), and are common among newborns with preterm birth (33, 34). Moreover, evidence suggests that preterm infants with higher BDNF levels have lower odds of failing in cognitive, motor, and social developmental domains (35). This association between BDNF and developmental outcomes is relevant considering the wide variety of functions that this neurotrophin has in brain and neurovascular development (36). In the central and peripheral nervous system, BDNF is abundantly expressed and it has a crucial role in neuronal growth and differentiation, as well as in synaptic plasticity of the developing fetus (37). In addition, BDNF has an important role in establishing neurogenesis and maintaining brain homeostasis. BDNF also has the potential to attenuate the neurotoxic effects of inflammation in the brain (38). This is essential considering that preterm birth is associated with increased neuroinflammation, which is an underlying mechanism for neuronal damage (39). All these functions posit BDNF as a main neurotrophin associated with neuroprotection and neurodevelopment particularly with cognitive and motor maturation (11). Our meta-regression model also revealed that BDNF levels are higher in placental tissue and cerebrospinal fluid than in peripheral blood and umbilical cord blood samples. This finding has implications for estimates of heterogeneity between studies; moreover, it is also important considering that BDNF plays an essential role in placental development and maturation (40). Future studies addressing the underlying molecular mechanisms implicated in reduced BDNF levels among preterm neonates are required.

Although we found consistent results for BDNF, our finding of lower NT-3 levels among preterm newborns should be carefully interpreted. Only a few studies contributed with effect size estimates, and therefore, we cannot rule out the possibility of lower accuracy in this analysis. Despite that, some studies have suggested that both NT-3 and BDNF may be the most sensible NFs with altered expression following gestational complications (41, 42), such as those associated with preterm birth. For instance, NT-3 and BDNF have been suggested as important neurotrophins for the regulation of angiogenesis and vessel maintenance during embryogenesis (41). In addition, NT-3 expression has been documented in placental tissue during different gestational trimesters, hence emphasizing its function in fetal and placental development (42).

The investigation of the other NFs, such as NGF and NT-4 did not reveal any significant effects. However, we did not rule out the possibility that preterm newborns might also have altered expression and production of these NFs, since our meta-analyses were performed with a small number of studies, which highlights the under exploration of some NFs. Interestingly, in some of the reviewed studies, we found relevant clinical correlations between NGF levels and neurodevelopmental outcomes (25, 29). Suggesting that these NFs should be further explored. In addition, although we did not perform meta-analysis for GDNF, Rajkumar et al. (28) found that blood GDNF levels were significantly higher in preterm newborns when compared to term newborns. In light of that, it is clear that more extensive research should be performed to investigate whether there is a relationship between disturbances in NGF, NT-4, or GDNF, and preterm birth.

To our knowledge, this is the broadest systematic review with meta-analysis and meta-regression of this subject to date. However, some limitations should be addressed. Our meta-analyses sample size was small considering the requirements to identify moderators of heterogeneity in meta-regressions. For NGF, NT-3, and NT-4, the sample size was even smaller. Thus, further investigation using larger sample sizes are needed to improve the assessment of methodological and sample variables that may explain the considerable heterogeneity observed between studies. Evidence suggests that nutritional intake, fetal and neonatal anthropometric indices (e.g., FGR, neonatal BMI), as well as neonatal neurophysiological data (e.g., visual and somatosensory evoked potentials, EEG) could be related to different neurodevelopmental outcomes among preterm newborns, including structural and functional brain markers (43). Moreover, additional sources of heterogeneity could not be addressed in our review because few studies reported on these data. Future meta-analysis studies should investigate Apgar score, sex, and other sources of heterogeneity when such data becomes available. The publication bias could not be adequately assessed through Egger's regression test, except for BDNF analysis, due to the small number of studies available. However, we believe that our search strategy was indeed comprehensive, and we analyzed each study utilizing the NOS. This meta-analysis was carried out with studies focusing on NFs assessed among newborns. However, some evidence suggests that the assessment of NFs in blood samples from the mothers could also predict the consequences of preterm birth among neonates (17). We also identified that some NFs were under investigated (as example of NGF and NT-4) or even non investigated (as the case of CDNF and MANF), which highlight an open field for further studies.

Finally, it is essential to highlight that even though we documented that preterm birth is associated with reduced BDNF and NT-3 levels, there is no defined reference range for the levels of these NFs, suggesting that more studies are needed to better clarify the clinical utility of the current findings (12). Despite that, given that a balanced expression of NFs is crucial for normal pregnancy progression (34) and, later, for adequate neurodevelopment of the children, the findings of this meta-analysis are promising. This is particularly relevant considering that alterations in the concentration of these neurotrophins may lead to various peri- and post-natal complications (37), such as an increased risk for neurodevelopmental disorders. These adverse outcomes could impact both cognitive and behavioral functioning and lead to psychosocial and clinical problems later in life.

## Data Availability Statement

The original contributions presented in the study are included in the article/Supplementary Material, further inquiries can be directed to the corresponding author/s.

## Author Contributions

FK, BS, and BK-S performed all the first steps of the systematic review (identification, screening, eligibility, and data extraction) and risk-bias analyses. ST and TV reviewed and solved any discrepancy in this first steps and risk-bias analyses. KC, BK-S, and TV performed the meta-analysis and created the plot and figures for data representation. LX and WW cooperated with their expertise in biomolecular research for development of data discussion. FK and BS were the major contributors in writing and TV, ST, and BK-S revised the final version of the manuscript. All authors declared that they read and approve manuscript final version.

## Conflict of Interest

The authors declare that the research was conducted in the absence of any commercial or financial relationships that could be construed as a potential conflict of interest.

## Supplementary Material

Supplementary Figure 1 Flow chart of the systematic review.
