# Supplementary material for: Neurotrophic Factor Levels in Preterm Infants: A Systematic Review and Meta-Analysis
Source: Front Neurol. 2021 Apr 1;12:643576. doi: 10.3389/fneur.2021.643576 (PMC8047113; doi:10.3389/fneur.2021.643576)
Supplement: Supplementary Figure 1 — Flow chart of the systematic review. [file Data_Sheet_1.PDF]

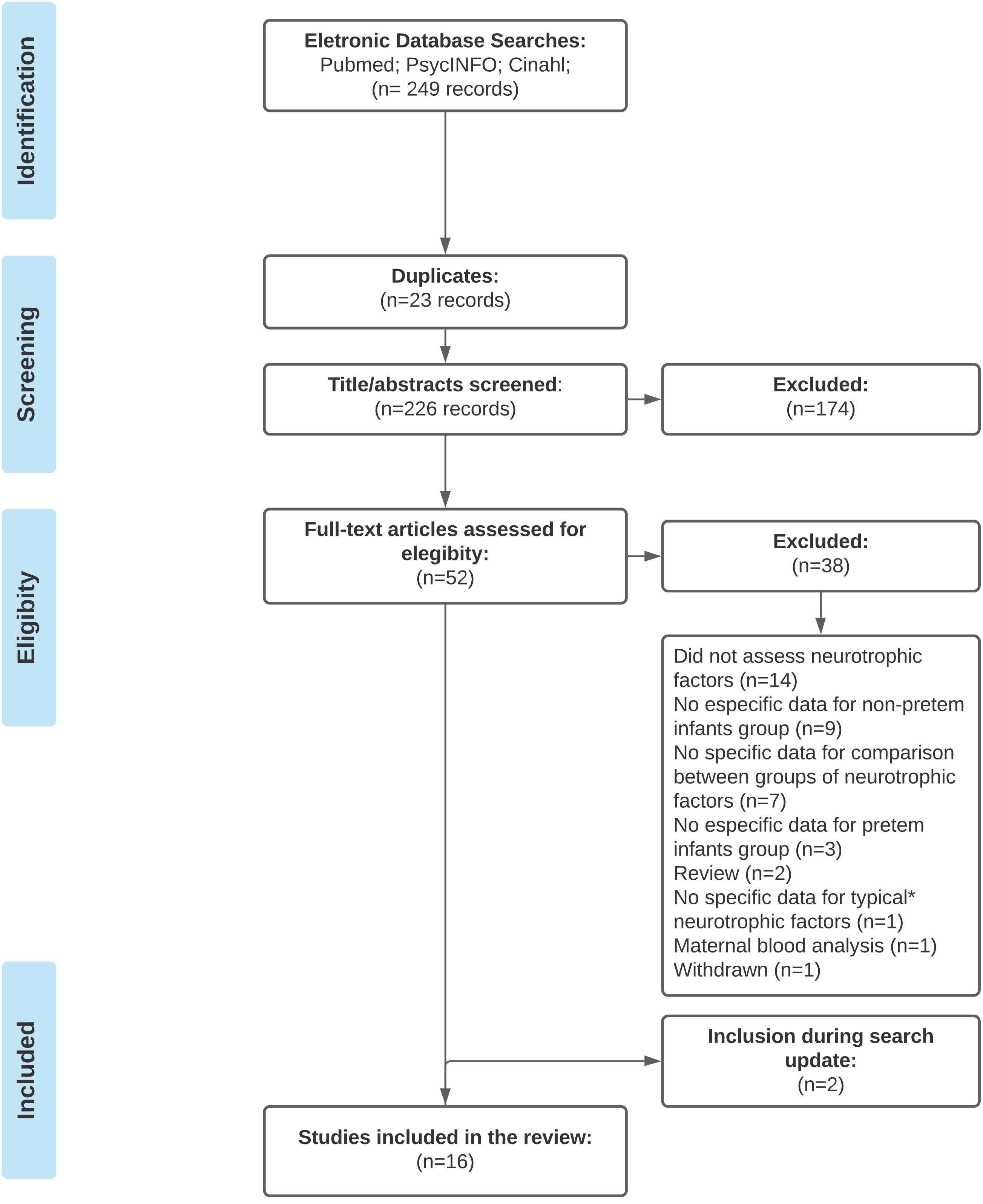
Identification
Screening
Eligibity
Included
Eletronic Database Searches:
Pubmed; PsycINFO; Cinahl;
(n= 249 records)
Duplicates:
(n=23 records)
Title/abstracts screened:
(n=226 records)
Excluded:
(n=174)
Full-text articles assessed for elegibity:
(n=52)
Excluded:
(n=38)
Did not assess neurotrophic factors (n=14)
No especific data for non-pretem infants group (n=9)
No specific data for comparison between groups of neurotrophic factors (n=7)
No especific data for pretem infants group (n=3)
Review (n=2)
No specific data for typical* neurotrophic factors (n=1)
Maternal blood analysis (n=1)
Withdrawn (n=1)
Inclusion during search update:
(n=2)
Studies included in the review:
(n=16)
